# Upgrading treatment strategies of cardiovascular diseases using biotechnology: Are we still there?

**DOI:** 10.34172/jcvtr.2021.19

**Published:** 2021-02-16

**Authors:** Sara Pahlavan, Sahand Abrishami

**Affiliations:** ^1^Department of Stem Cells and Developmental Biology, Cell Science Research Center, Royan Institute for Stem Cell Biology and Technology, ACECR, Tehran, Iran; ^2^Department of Cardiovascular Surgery, Imam Khomeini Hospital Complex, Tehran University Medical Center, Tehran, Iran

Two decades of hopes and failures in search for next generation therapeutics of cardiovascular diseases, is coming to an end by approaching 2021. The beginning of 21st century was coincided with a burst of hopes toward introduction of novel cell-based therapies for damaged myocardium. Therapeutics which could surpass the intermittent satisfactory reflow and/or revascularization therapies. By far, one can subdivide such preclinical attempts and clinical trials into two categories; those attempted repair and those aimed regeneration.^1,2^ The bone-marrow derived cells including unfractionated cells or mesenchymal stem cells could reside in the first category along with skeletal myoblasts and adipose-derived cells. Although these cells have not undergone trans-differentiation into cardiomyocytes after transplantation, but could improve cardiac function through their high secretory profile and mostly by immunomodulatory and proangiogenic effects.^2^ This can explain their promising impacts, despite extremely low engraftment degree ^3^ or other possible issues such as ageing and host tissue response. Thus, these sorts of cell therapy could have just been an adjuvant to the common reperfusion or coronary bypass graft procedures.^1^ In the context of regeneration as the second category, however, scientific community hoped to have the privilege of remuscularizing damaged myocardium. This attempt has started by using cKIT^+^ cells as the resident progenitor cells of the heart as well as cardiosphere-derived cells.^2^ However, lineage tracing studies showed that cKIT^+^ cells mainly differentiated into non-myocytes cells of the heart,^2,4^ disproving their remuscularization capacity. The introduction of *in vitro* generated human cardiomyocytes from directed differentiation of pluripotent stem cells (hPSC-CM) brought some new hope in the context of remuscularization. While the main goal of this approach was regeneration, concerns on the possible arrhythmogenic events were hindered its application. Bottom-line, this hPSC-CMs are now being tested in a doctor-initiated clinical trial (jRCT2053190081). Clinical trials which were performed for cell therapy of cardiovascular diseases, in general, nor induced considerable cardiac repair, neither regeneration, but just neutral or marginal improvement. Two decades of preclinical experiments and clinical trials, however, taught us to first resolve the following issues before taking any further actions:

1. Clearly identifying the desired therapeutic outcome; cardioprotection or regeneration
2. Choosing optimal cell type preclinically
3. Selecting the best route of administration and developing tractable delivery systems
4. Identifying long-term safety and efficacy in preclinical large animal models
5. Establishing general regulations for more consistent and rigorously designed clinical trials with randomized, adequately powered, placebo-controlled, blinded arrangements and evaluations based on clinically relevant markers
6. Selecting ideal patient subsets
7. Research and development toward good manufacturing practice (GMP) of advanced therapeutic medicinal products (ATMP) in order to have scalable clinical grade products and reduce batch-to-batch differences.

It is worth noting that apart from cell therapy, previous experiences led into other biotechnology-related products (i.e. everything related to the biology of the cells that can be harvested or synthesized by advanced technology) as candidates for cardioprotection or regeneration. Extracellular vesicles which are membrane-enclosed entities secreted from cells, with a cargo of specific proteins, small RNAs and other signaling molecules, have emerged as promising therapeutics for cardiovascular diseases. Depending on their cells of origin, they can induce immunomodulatory, anti-apoptotic, anti-fibrotic and proangiogenic effects^2^. While they lack direct remuscularization capacity, they might deliver regenerative signals to myocardial resident cells. As another potential candidate, gene therapy can be pursued for cardiovascular therapeutics. Three decades of introducing gene therapy products, did not include a successful candidate for heart. However, there is a big opportunity here and that is exploring candidate genes which either participate in cardioprotection or regeneration.^5^ For cardioprotection, genes underlying important proteins of immunomodulation, anti-apoptosis, anti-fibrosis and pro-angiogenesis can be targeted. On the other hand, genes involved in cell cycle reentry or redox regulation can be targeted for regeneration. Last but not least, replacement of RNA therapeutics for gene therapy, as a more direct modulator of gene expression by means of microRNAs or long non-coding RNAs, and most recently modRNAs, opened a new avenue in the research toward biotechnology-related therapeutics of cardiovascular diseases.^6^ Although, the development of next generation therapies for heart has been fairly slow, future would definitely celebrate the emergence of such products for a healthy heart life.
